# Cellular Solutions: Evaluating Single-Cell Proteins as Sustainable Feed Alternatives in Aquaculture

**DOI:** 10.3390/biology14070764

**Published:** 2025-06-25

**Authors:** Korale Kankanamge Dinuka Chamodi, Nguyen Thanh Vu, Jose A. Domingos, Jiun-Yan Loh

**Affiliations:** 1 Aquaculture and Fisheries Group, Wageningen University and Research, Droevendaalsesteeg 4, 6708 PB Wageningen, The Netherlands; dinukachamodi2017@gmail.com; 2 Animal Sciences and Aquatic Ecology, Faculty of Bioscience Engineering, Ghent University, Sint-Pietersnieuwstraat 25, B-9000 Ghent, Belgium; 3 Tropical Futures Institute, James Cook University, 149 Sims Drive, Singapore 387380, Singapore; vu.nguyen2@jcu.edu.au (N.T.V.); jose.domingos1@jcu.edu.au (J.A.D.)

**Keywords:** alternative protein, biofortification, circular economy, nutrigenomics, single-cell protein

## Abstract

The rapid expansion of the global population has intensified the demand for protein-rich food sources, positioning aquaculture as a crucial sector in the endeavor to alleviate global hunger through the provision of high-quality aquatic protein. Traditional protein sources such as fishmeal have historically served as the foundation of aquafeeds; however, their elevated costs and limited availability have catalyzed the search for sustainable alternatives. These alternatives encompass plant-based proteins, insect meals, and, more recently, single-cell proteins (SCPs), which are derived from microorganisms including bacteria, yeast, fungi, and microalgae. Nonetheless, SCP remains in its nascent stages and currently accounts for only a minor fraction of aquafeed formulations relative to other established alternatives. The production of SCP utilizes low-cost substrates, such as agricultural and dairy wastes, thereby supporting waste mitigation and principles of the circular economy. This review elucidates the nutritional value of SCPs, their potential for biofortification, and their emerging roles as functional feeds with immunomodulatory and nutrigenomic effects. Additionally, the review underscores the potential of endophytes as a novel SCP source, highlighting their underutilized capacity to foster sustainable innovations in aquafeeds.

## Simple Summary

Single-cell proteins (SCPs) are emerging as promising alternative protein sources in aquaculture, offering sustainable solutions to meet the rising demand for fish feed. This review explores various SCP sources globally, including microalgae, bacteria, yeasts, fungi, and endophytes, with a focus on their nutritional benefits and functional properties. Evidence suggests SCPs enhance fish health, immunity, and growth performance. Endophytes, in particular, show potential due to their bioactivity and high protein content. However, despite their advantages, challenges such as production costs, scalability, and consumer acceptance hinder widespread adoption. This paper also outlines future directions to improve SCP integration in aquafeeds.

## 1. Introduction

The global population is anticipated to reach approximately 8.5 billion by the year 2030, precipitating a heightened demand for food resources [1,2]. In 2023, over 735 million individuals globally were reported to experience undernourishment, with the highest prevalence occurring in South Asia and Sub-Saharan Africa, thereby underscoring significant regional disparities in food security [3]. Ensuring the provision of adequate food to satisfy the needs of an expanding global population continues to present a critical challenge. Aquaculture, recognized as the fastest-growing food production sector, plays an indispensable role in addressing this challenge [4]. Fish, a fundamental component of global nutrition, offers high-quality protein, n-3 polyunsaturated long-chain fatty acids (PUFAs) such as eicosapentaenoic acid (EPA) and docosahexaenoic acid (DHA), along with essential micronutrients including iodine, selenium, calcium, iron, and zinc [5,6].

Global aquaculture production of aquatic animals is projected to reach approximately 111 million tonnes by 2032, reflecting its pivotal role in meeting future food demands [2]. In 2022, aquaculture production of aquatic animals surpassed that of capture fisheries for the first time, yielding 94.4 million tonnes, with 57% allocated for human consumption [2]. However, the growth of aquaculture is impeded by challenges in feed formulation, particularly the substantial reliance on fishmeal as a primary aquafeed ingredient [7].

Fishmeal, esteemed for its high protein content, balanced essential amino acid (EAA) profile, superior nutrient digestibility, absence of anti-nutritional factors (ANFs), affordability, and consistent availability, remains a cornerstone of aquafeeds [8,9]. Global fishmeal production is projected to increase by 9% by 2032 compared to 2022 [2]. Peru, as the leading producer of fishmeal, plays a significant role in the global supply chain. However, forecasts based on U.S. Department of Agriculture data suggest a 4% decline in Peruvian fishmeal production by 2028. Sustainability concerns are heightened due to its dependence on wild-caught small pelagic fish, such as anchovies and sardines, which are susceptible to overexploitation, climatic variations, and market volatility [10,11].

Efforts to enhance sustainability have led to an increased utilization of fishmeal derived from by-products, such as fish carcasses, trimmings, and offal, a trend expected to expand by 2032 [2]. Nevertheless, international fishmeal prices rose sharply from USD 452 per tonne in 2000 to USD 1596 per tonne in 2018, with further increases expected due to continued strong demand [11]. These escalating costs significantly contribute to higher production expenses in aquafeeds, which constitute approximately 50% of aquaculture production costs [12,13].

Ideal fishmeal alternatives should provide a balanced amino acid profile, high digestibility, low fiber and carbohydrate levels, competitive pricing, minimal environmental impact, and ease of incorporation while being readily accessible [9]. Protein sources derived from plants and animals have been investigated as substitutes for fishmeal, with inclusion levels ranging from 10% to 80% for plant-based proteins and 2% to 100% for animal-based proteins, either partially or fully replacing fishmeal [9]. These alternatives exhibit varying degrees of efficacy in achieving a balance between sustainability and nutritional adequacy. Innovations including plant-based proteins, insect meals, algae meals, and single-cell proteins (SCPs) have emerged as viable alternatives [14,15,16,17]. Among these, SCPs are distinguished by their nutritional, environmental, and economic benefits [18].

SCPs refer to proteins derived from microorganisms such as bacteria, yeasts, fungi, or algae, cultivated on a large scale for utilization as nutritional sources in both human and animal diets [19] as well in fish diets [20] (Table 1). The term “single-cell protein” was first introduced by Professor Carroll Wilson at the Massachusetts Institute of Technology (MIT) in 1966 to supplant the less appealing terms “microbial protein” and “petroprotein” [21,22]. It is also known as bioprotein or microbial biomass [23]. Microbial biomass can be classified based on its primary composition into SCP or single-cell oil (SCO) resources. SCP typically contains over 300 g/kg protein (dry weight), while SCO refers to oleaginous products with lipid content exceeding 200 g/kg [24].

The incorporation of microorganisms into human diets is a longstanding practice, as these entities have been consumed for centuries in various foods, including cheese, yogurt, soy sauce, and alcoholic beverages, whether through intentional inclusion or incidental consumption [29]. The origins of this practice can be traced back to 2600 BC in Babylonia, where the earliest evidence of bread making was documented. By the time of Hammurabi in the 12th century BC, the craft of baking had evolved into a specialized trade [21]. Algae have historically been consumed, while bacteria such as *Lactobacillus* and *Acetobacter* are frequently ingested inadvertently through numerous food and beverage items [29]. According to [30], the concept of single-cell protein (SCP) emerged during the two World Wars as a crucial strategy for mitigating protein shortages. In World War I, *S. cerevisiae* (yeast) was cultivated on molasses to substitute up to 60% of imported protein and fossil fuel needs in Germany. During World War II, *Candida utilis* (yeast) was produced from paper waste to assist in addressing protein deficiencies among starving populations [30].

In the 1970s, SCP products such as Pruteen, a bacterial single-cell protein, were developed as a prospective solution to future food crises; however, the increase in methane prices during the 1980s oil crisis resulted in the cessation of its production. The Pruteen facility produced a high-protein livestock food supplement by dehydrating a microbial biomass of *Methylophilus methylotrophus*, cultivated on methanol as the sole carbon source [31]. Quorn Foods has utilized submerged fermentation to produce mycoprotein for consumers since 1985, initially in the United Kingdom and later expanding to the United States [32]. In 2018, the company aimed to significantly enhance its production capacity to achieve an annual output of 40,000 tonnes [33]. The growing recognition of the global protein supply–demand disparity, exacerbated by an increasing world population, has reignited interest in SCP production over the past five decades [30].

SCPs, sourced from microbial biomass, provide distinctive advantages over traditional protein sources [34]. They are extensively utilized across various sectors, serving as ingredients in animal feed and in the food industry for meat substitutes, dairy alternatives, snacks, cereals, and beverages [35]. SCPs are high in protein, can proliferate rapidly using low-cost substrates with minimal soil, water, and specific temperature requirements, and, unlike plant-based proteins, are not affected by seasonal or climatic fluctuations while necessitating minimal land use [21,34,36,37,38]. The production of SCP from agro-waste aligns with global sustainability goals under Sustainable Development Goal (SDG) 12, addressing challenges related to food loss and waste [39]. Agricultural residues, such as fruit peels and sugarcane bagasse, have been investigated as substrates, rendering SCPs cost-effective and environmentally sustainable [40].

**Figure 1:**
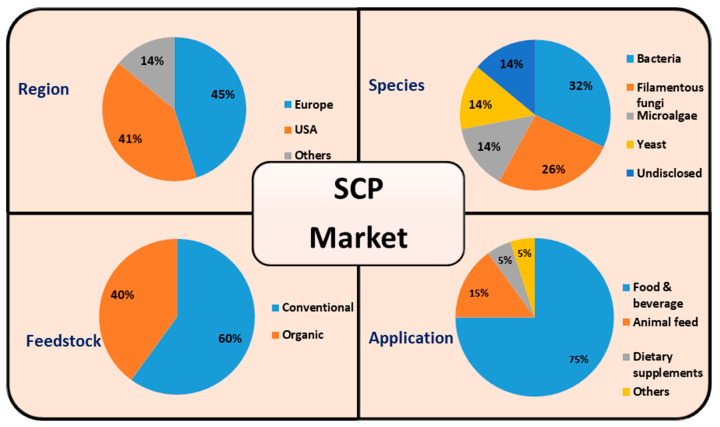
SCP market segmentation. Modified based on [29,43].

The global market for Single Cell Proteins (SCPs), which was valued at USD 10.36 billion in 2023, is projected to grow to USD 22.49 billion by 2032 [41]. This growth is driven by rising demand for sustainable protein alternatives across food, animal feed, and industrial sectors [41]. SCP production is categorized by species (yeast, bacteria, algae, fungi), feedstock types (conventional and organic), and applications, with food and beverages comprising 75% of the market share in 2022 [42,43] (Figure 1). Regionally, North America and Europe lead the market due to advanced research infrastructures and robust sustainability initiatives, while the Asia-Pacific region is experiencing rapid growth due to population expansion and increasing protein demand [41]. Algae and fungi are the predominant species utilized in SCP production, particularly for food applications. Approximately 60% of SCP production is reliant on conventional feedstocks, although organic feedstocks are gaining momentum, reflecting a 4% increase in adoption due to sustainability concerns. The human consumption sector accounts for 59% of SCP utilization, while 41% is employed in animal feed [30].

Despite its potential, the application of SCPs in aquafeeds remains underutilized [44]. SCPs are predominantly employed as supplementary feed additives rather than as primary protein sources [20]. The predominance of a limited number of countries, such as Peru and Chile, in global fishmeal production, combined with challenges such as overexploitation of fishery resources, inconsistencies in availability, and price volatility, underscores the urgent necessity for sustainable alternatives such as SCPs [45,46]. Research into the nutrigenomic and immunomodulatory effects of SCP remains limited.

This review focuses on the niche applications of SCPs in aquaculture, emphasizing their nutritional value and potential for biofortification, particularly from emerging sources such as endophytes. It examines their role in modulating immunity, enhancing growth, improving feed intake, and promoting gut health, thereby positioning them as functional feed ingredients. Furthermore, it investigates the role of nutrigenomics in elucidating the molecular mechanisms that contribute to the benefits of SCPs. By addressing these factors, this review aims to provide novel insights into the application of SCPs in sustainable aquafeed development and the reduction of reliance on traditional fishmeal sources.

## 2. SCP Market

The SCP industry is characterized by advancements in microbial protein technologies developed by established companies, alongside scalable, eco-friendly solutions offered by startups. Investments in research, innovation, and collaborative partnerships are propelling growth, positioning SCP as a crucial element of sustainable protein development across various regions and applications.

As of 2023, approximately 35 companies have entered the market with commercially available SCP products derived from diverse microorganisms, including yeasts, fungi, microalgae, and bacteria, with the United States and Europe at the forefront of production [47]. Calysta is a leader in gas fermentation technology and has successfully established the world’s first commercial FeedKind production facility in Chongqing, China, in partnership with Adisseo, producing sustainable protein products for animal feed [48]. Calysta’s FeedKind Aqua^®^ (Calysta, San Mateo, CA, USA) is a versatile, fermented, bacteria-based protein that enhances gut health in aquaculture species such as shrimp, salmon, and warmwater carnivorous finfish, promoting digestive health and immunity [49,50]. In shrimp, it has been demonstrated to strengthen the immune system and diminish susceptibility to *Vibrio*, which is associated with early mortality syndrome (EMS).

The Commonwealth Scientific and Industrial Research Organisation (CSIRO) developed a product named NovacqTM using marine microbes. Prawns fed NovacqTM exhibit accelerated growth and improved health with reduced reliance on wild fish in their diets [51,52]. KnipBio Meal (KBM) has achieved a significant milestone, becoming the first premium single-cell protein to be granted Generally Recognized As Safe (GRAS) status by the U.S. Food and Drug Administration (FDA) Center for Veterinary Medicine, facilitating its use as an approved aquafeed ingredient for salmonids and various other finfish species [53]. KBM is a single-cell protein derived from *M. extorquens*, designed as a sustainable alternative to fishmeal in aquafeeds [54,55]. A summary of current countries and key companies producing SCP products is presented in Table 2. However, the cost of SCP is not yet competitive with soy protein or fishmeal, and large-scale production continues to pose a challenge [33,56].

## 3. Different Types of SCP

Various types of SCPs, including yeasts, fungi, microalgae, and bacteria, are utilized in aquaculture, each offering distinct advantages and limitations (Table 1). These SCPs are administered through various methods in aquaculture systems, such as direct inclusion in aquafeeds as feed ingredients, bioencapsulation, incorporation into bioflocs, supplementation in aquafeeds, and suspension in water.

### 3.1. Yeast and Other Fungi

Yeasts, which are rich in amino acids, B vitamins, and lysine, are widely used in animal feed. However, their low methionine and cysteine content limits their effectiveness as a sole protein source [23]. Yeast-based SCP has shown promise in aquaculture, particularly for its ability to enhance bile production and maintain acidic pH levels, thereby creating a safe environment free from antibiotic resistance genes [57,58]. Common yeast species used as sources of SCP include *S. cerevisiae*, *Kluyveromyces marxianus*, and *Candida utilis* [46,58]. Notably, methylotrophic yeasts like *C. utilis* can efficiently convert methanol into biomass and proteins [59]. Despite this, limited studies have focused on incorporating dried yeast as a primary protein source in aquaculture feeds, such as in salmon diets [46].

Yeast and fungal SCP derived from species such as *Aspergillus* sp., *Paecilomyces* sp., *Kluyveromyces* sp., *Candida* sp., and *Fusarium* sp. offer a nutrient-rich alternative. These species are valued for their high lysine, threonine, and vitamin-B-complex content, as well as folic acid [60,61,62]. Fungi and yeasts can achieve protein content levels of 30–50% under optimized fermentation conditions, making them promising candidates for SCP production. However, challenges such as high nucleic acid content (up to 10%) and low cell wall digestibility must be addressed before their full potential can be realized [36,63].

In aquaculture, yeast and fungal SCP are primarily included in aquafeeds to partially replace traditional protein sources. Species such as *S. cerevisiae*, *A. niger*, and *Penicillium variotii* have been studied in diets for *Cirrhinus reba* (reba carp), *Carassius auratus* (Hefang crucian carp), *Sparus aurata* (gilthead sea bream), and *Oncorhynchus mykiss* (rainbow trout) [61,64,65,66]. Similarly, brewer’s yeast has shown potential as an alternative to fishmeal in the diets of *Macrobrachium rosenbergii* (giant freshwater prawns) raised in either biofloc systems or recirculating aquaculture systems (RASs) [67]. While dietary inclusion is the predominant method for administering SCP, yeast SCP has also been used directly as feed through water. For example, rotifers have been cultivated using baker’s yeast [68], and *Scophthalmus maximus* (Turbot) were fed *Artemia* nauplii pre-enriched with baker’s yeast bioencapsulated in an oil emulsion [69].

### 3.2. Microalgae

Microalgae have emerged as promising alternatives in aquaculture feed due to their exceptional nutritional and functional properties. Some microalgae are rich in protein containing all essential amino acids, while others provide significant sources of essential fatty acids and bioactive compounds that contribute to health benefits [70]. The crude protein content in microalgal biomass typically ranges from 30% to 80% by mass [27]. Species such as *Chlorella*, *Spirulina* (Arthrospira), and *Dunaliella* are prominent in protein production, with protein contents of 55%, 65%, and 57%, respectively, making them commercially valuable [71].

Microalgae are widely used in aquaculture, primarily as food for live feed organisms or directly for small larvae [68]. For example, chlorophytes such as *Chlorella* and *Tetraselmis chuii* and chrysophytes like *Isochrysis* sp. are common in shrimp hatcheries, where they serve as essential feed for shrimp larvae [68]. Additionally, microalgae SCP can be delivered through the enrichment of zooplankton such as rotifers and *Artemia*, which are subsequently fed to larval fish and crustaceans [68,72,73]. The administration methods include offering live microalgae, dried powders, or concentrates via water, enabling direct consumption or enrichment of live prey organisms [68]. For instance, rotifers perform better nutritionally when enriched with *Chlorella* due to its high essential fatty acid (EFA) content [74]. Another approach involves microalgae encapsulation, which serves as a direct method for delivering SCP to aquatic species [75,76]. *Artemia* metanauplii were bioencapsulated with the microalgae *Chlorella minutissima* and *Tetraselmis chuii* to support the weaning process of Senegalese sole (*Solea senegalensis*) post-larvae [77].

Microalgae SCPs have also been incorporated into fish diets to replace fishmeal. Species such as *S. pacifica*, *S. platensis*, *C. vulgaris*, and *Scenedesmus almeriensis* have been used in feeding parrot fish (*Oplegnathus fasciatus*), Gangetic mystus (*Mystus cavasius*), African catfish (*Clarias gariepinus*), and gilthead sea bream (*S. aurata*) [78,79,80,81]. However, despite their potential, the high production cost of microalgae limits widespread application [68]. Beyond direct inclusion as feed ingredients, microalgae such as *Spirulina* sp. are widely utilized as feed supplements. For example, ref. [82] explored the effects of *Spirulina*-powder-supplemented feeds on the growth performance of abalone. The diverse applications of microalgae SCP in aquaculture highlight their critical role in reducing reliance on traditional fishmeal and enhancing sustainability in the industry.

### 3.3. Bacteria

Bacterial SCPs typically contain the highest protein concentrations among SCP sources, ranging from 50% to 80% on a dry weight basis [24]. Numerous bacterial species have been studied for their potential in SCP production [26]. Bacteria are highly suitable for SCP production due to their rapid growth, short generation time, high protein content (50–80%), efficient production processes, genetic adaptability, and well-characterized strains [38].

Bacteria assimilate protein through diverse metabolic pathways, thereby enhancing their potential for SCP production. Methanotrophy allows methanotrophic bacteria, such as *Methylococcus capsulatus*, to convert methane into biomass via the ribulose monophosphate (RuMP) or serine pathways, resulting in high protein yields while simultaneously reducing greenhouse gas emissions [83,84]. Photoheterotrophy, utilized by photosynthetic bacteria like purple non-sulfur bacteria (PNSB), harnesses light energy and organic carbon to assimilate nutrients through the tricarboxylic acid (TCA) cycle, facilitating efficient protein synthesis [26,85]. Furthermore, bacteria capable of lignocellulose degradation, such as *Rhodococcus opacus*, decompose plant biomass into fermentable sugars, which are subsequently metabolized into protein-rich biomass, providing a sustainable approach for SCP production utilizing agro-industrial residues [86,87].

Among various microorganisms, *Methylobacterium* species have recently garnered attention for their significant role in SCP production [38]. Bacterial protein derived from *M. capsulatus* through natural gas fermentation presents a valuable protein source, characterized by a favourable amino acid composition and digestibility, thus supporting animal growth and health, with potential improvements achievable through enhanced processing techniques and nutrient utilization research [83]. Additionally, PNSB exhibit a protein content of 70–72% and demonstrate high resistance to toxic substances, with their amino acid profile closely resembling that of soybean protein [26]. *R. opacus* shows promise for SCP production by utilizing agro-wastes as cost-effective substrates [86]. Products derived from bacterial SCP offer enhanced phosphorus availability compared to traditional fishmeal sources [88].

Bacterial SCP plays a crucial role in BFT systems, where bacteria are the predominant microorganisms and serve as a protein source for aquatic species [89]. Bacterial SCP can be administered to aquatic animals through bioencapsulation techniques, which involve creating a protective layer around individual probiotic cells [90]. Bioencapsulated probiotics have demonstrated significant potential for enhancing the growth and survival rates of juvenile fish species [91]. In aquaculture, bioencapsulation methods are commonly employed to deliver probiotics to species such as whiteleg shrimp (*Penaeus vannamei*) and rainbow trout (*Oncorhynchus mykiss)* [92,93,94,95].

Recent studies have integrated methanotrophic bacterial SCPs into aquafeeds as a substitute for fishmeal, administering them orally in the diets of rainbow trout (*O. mykiss*) and barramundi (*Lates calcarifer*) [17,96]. SCP derived from *M. capsulatus* has been incorporated into the diets of Pacific white shrimp (*P. vannamei*) [97].

Bioflocs, another source of bacterial SCP, have gained recognition for their ability to replace fishmeal or soybean meal in aquaculture feeds. BFT involves microbial communities composed of bacteria, algae, and protozoa, collectively producing bioflocs rich in proteins and lipids [98]. Bacteria are often included in BFT systems, where the nutritional advantages of bioflocs encompass reduced dietary protein requirements and the potential to substitute 30% to 50% of fishmeal in diets for farmed fish and shrimp [98,99]. Delivery methods include the use of in natura (wet) biofloc biomass or dry biofloc meal (BFM) as direct feed ingredients [98].

Bacterial SCP represents a versatile and sustainable protein source in aquaculture, offering high nutritional value and efficient production. Its applications range from direct dietary inclusion to probiotic delivery and integration within BFT systems, providing a sustainable alternative to fishmeal and supporting environmentally friendly aquaculture practices.

### 3.4. Endophytes as a Potential Source of SCP

**Figure 2:**
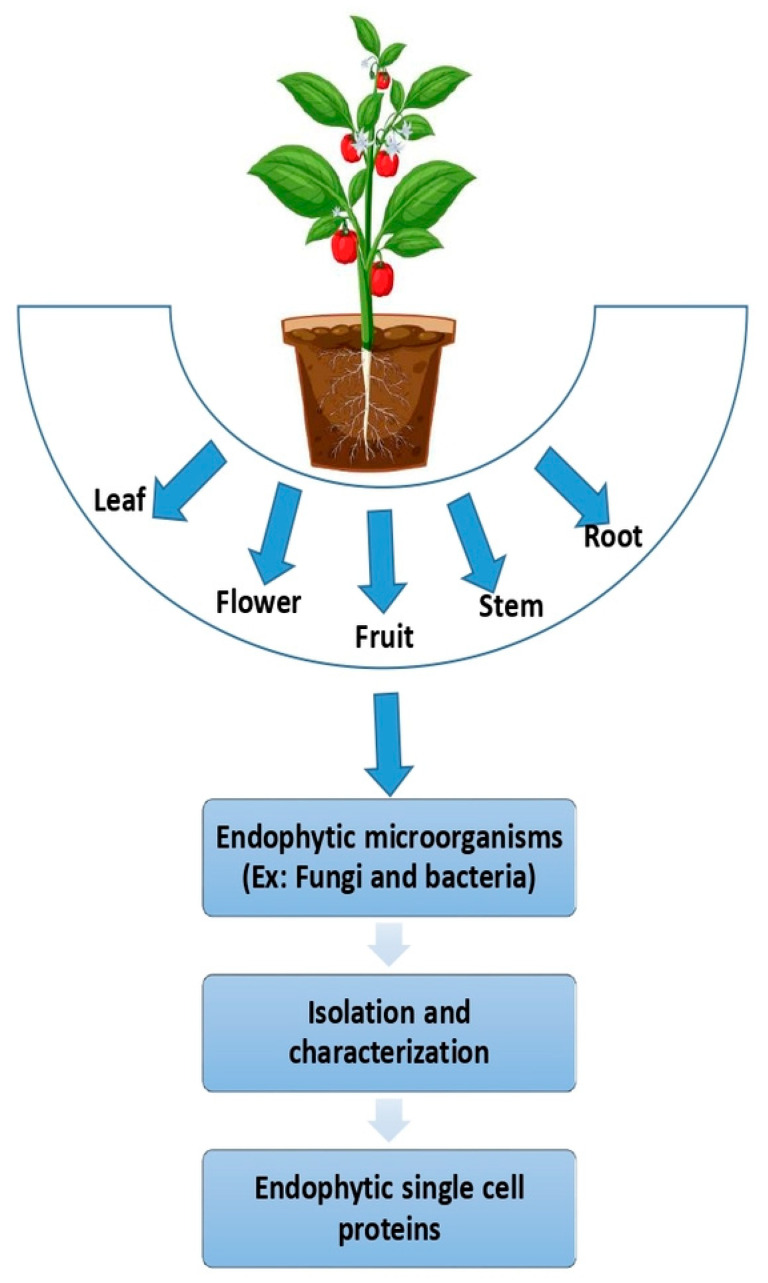
Extraction of endophytic SCPs from a plant.

Endophytes, encompassing bacteria and fungi, are microorganisms that establish symbiotic relationships within plant tissues (including leaves, flowers, fruits, stems, and roots), often occupying intercellular or intracellular spaces [100] (Figure 2). These microorganisms inhabit plant tissues without inducing disease symptoms and contribute to host growth by enhancing nutrient acquisition, improving resistance to environmental stresses, and providing protection against pests [101]. They are prevalent in both terrestrial and aquatic plants, including algae and seaweed, where they contribute to plant health and can exhibit biological activity against pathogens [102,103].

Fungal endophytes, in particular, have been extensively studied due to their ecological importance and diversity, including groups such as ascomycetes, basidiomycetes, and anamorphic fungi [104,105]. Notable examples of fungal endophytes include *Penicillium chrysogenum*, *Fusarium* species, and *Paracamarosporium leucadendri* [106]. These fungi serve as important sources of bioactive compounds, such as antibiotics and anticancer agents [101]. For instance, penicillenols from *Penicillium* species exhibit cytotoxic effects, while Taxol from *Taxomyces andreanae* is a well-known anticancer drug derived from endophytic fungi [101].

Yeasts also represent a group of endophytic microorganisms recognized for their ability to rapidly colonize plant surfaces and form biofilms, which protect plants from pathogens by inhibiting mycelial growth and spore production [107]. Thriving in diverse environments, yeasts utilize available nutrients and exhibit resistance to temperature variations, rendering them valuable for various applications [107,108].

Additionally, bacterial endophytes, such as *Pseudomonas* sp., *Staphylococcus* sp., *Azotobacter* sp., *Enterobacter* sp., *Serratia* sp., *Clavibacter* sp., and *Bacillus* sp., are associated with a wide variety of plant species and belong to predominant phyla such as Actinobacteria, Proteobacteria, and Firmicutes [109,110]. Actinomycetes, a subgroup of bacterial endophytes within the phylum Actinobacteria, are recognized for their production of secondary metabolites with antimicrobial, antitumor, and other therapeutic properties [111]. These actinomycetes also produce antimicrobial compounds, including alkaloids, peptides, and flavonoids, as well as industrially valuable enzymes such as cellulase, amylase, and protease [111,112].

Given their ability to produce bioactive compounds, endophytes are emerging as promising sources of SCP. Studies have demonstrated the capacity of endophytic fungi isolated from bamboo, such as *Cladosporium cladosporioides*, *A. niger*, *A. flavus*, *Penicillium citrinum*, *Monascus ruber*, and various *Fusarium* species, to produce SCPs through solid-state fermentation on substrates such as rice bran and corn cobs [113,114]. Furthermore, *Methylobacterium extorquens*, an epiphytic bacterium, has been identified on plants such as *Rosa* sp., garden strawberry (*Fragaria × ananassa*), and *Hibiscus* sp. Its presence has been shown to trigger plant defense mechanisms, thereby enhancing the synthesis and storage of antimicrobial compounds [115,116,117]. This bacterium could also be explored as an endophyte with potential as a source of SCP in aquaculture. By isolating and cultivating *M. extorquens* as an endophyte, it could be developed into a sustainable SCP source for aquafeeds, providing a nutritious and eco-friendly alternative to conventional protein ingredients while supporting the growth and health of aquatic species.

## 4. Nutritional Value of SCP

The nutritional value of SCP is contingent upon the type of microorganism employed, as well as the harvesting, drying, and processing methods utilized, all of which can significantly influence the nutritive quality of the final product [118]. The substrate used for its cultivation also affects the nutrient composition of the final SCP product [118]. SCP is highly regarded for its rich nutrient composition [21]. These microorganisms not only provide proteins but also offer free amino acids, carbohydrates, nucleic acids, lipids, vitamins (B1, B2, B3, B5, B6, B7, B9, B12, C, E, and β-carotene), and essential minerals such as phosphorus and potassium [63,119].

Microalgal SCPs serve as a nutrient-dense source, offering nucleic acids, essential minerals, and vitamins such as A, B-complex, C, D, and E, alongside amino acids like leucine, valine, lysine, and phenylalanine [71]. They contain elevated levels of riboflavin, thiamine, folic acid, and carotene [120]. *Spirulina* and *Chlorella* are recognized sources of vitamin B12 [120]. Microalgal SCPs, including *Spirulina maxima* (up to 71%), *Chlorella* sp. (up to 58%), *Scenedesmus obliquus* (up to 56%), and *Scenedesmus acutus* (up to 64%), are notable for their high protein content [63].

Fungi-based SCP comprises approximately 50–55% protein, is rich in lysine, exhibits a high protein-to-carbohydrate ratio, possesses a balanced amino acid profile, and is abundant in B-complex vitamins [118]. Yeast serves as a valuable source of essential nutrients, including B vitamins such as thiamine, riboflavin, and niacin, as well as biotin, folic acid, glutathione, and p-amino benzoic acid, which are critical for various biological functions [121]. The yeast cell wall, an integral structural component, is primarily composed of β-glucan (29–64%), mannan (31%), chitin (1–2%), proteins (13%), and lipids (9%), collectively accounting for 25–30% of the cell’s dry weight [122,123]. The chemical composition of yeast species is influenced by their growth substrate. For example, *S. cerevisiae* biomass produced through fermentation on starch powder had a higher crude protein content (506.3 g/kg DM) than that produced on chili stubble (389.0 g/kg DM), demonstrating a significant effect of substrate on nutritional quality [124]. Furthermore, *S. cerevisiae* efficiently metabolizes hexose sugars, while other species specialize in fermenting pentose sugars [122]. Bacterial-derived SCP can offer protein levels of up to 80% on a dry weight basis, with an amino acid composition comparable to that of fishmeal or soybean protein, in addition to a high concentration of vitamins, phospholipids, and other beneficial compounds [38].

## 5. Enhancement of the Nutritional Value of SCP

Biofortification is a strategy employed to enhance the nutrient content of food, commonly applied in crop production and increasingly explored in aquaculture to improve the nutrient profile of aquatic organisms [125]. Various studies have demonstrated the potential of biofortification in improving the nutritional quality of aquafeeds. For example, iodine-rich sugar kelp has been incorporated into rainbow trout feed formulations to increase iodine content [126]. Similarly, the incorporation of the red macroalga *Asparagopsis taxiformis* into juvenile white seabream diets has been shown to enhance performance, even under heat stress conditions [127]. Other studies, such as those conducted by [128], explored the biofortification of gilthead seabream diets with *Laminaria digitata*, resulting in improvements in immunomodulatory and antioxidant responses. Additionally, ref. [129] evaluated diets containing selenized yeast and macroalgae, which enhanced growth, nutrient utilization, and immune gene expression in gilthead seabream.

**Figure 3:**
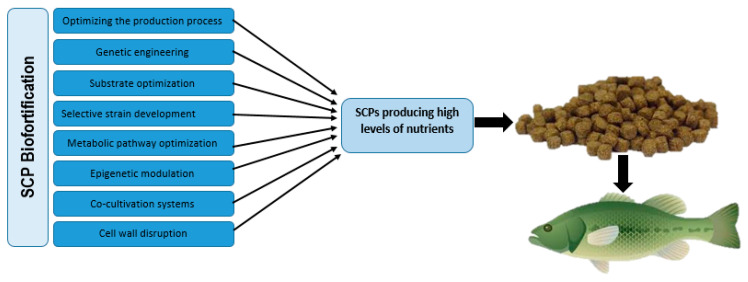
Strategies for SCP Biofortification to Enhance Nutritional Quality in Aquafeeds.

Moreover, SCPs present a promising opportunity for biofortification due to their capacity to deliver essential nutrients for fish health. Several approaches are employed to enhance the nutritional value of SCPs, including optimizing the production process, genetic engineering, substrate optimization, selective strain development, metabolic pathway optimization, epigenetic modulation, co-cultivation systems, and cell wall disruption (Figure 3). For instance, the application of selenized yeast to biofortify the nutritional quality of *Sparus aurata* and *Cyprinus carpio* demonstrates the significant role of selenium in supporting fish growth and physiological functions [130,131]. The biofortification of SCPs through these methodologies not only enhances their nutritional profile but also serves to mitigate the impacts of environmental stressors, a critical factor for sustainable aquaculture.

### 5.1. Optimizing the Production Process

The optimization of culture conditions, including temperature, pH, nutrient availability, and oxygen levels, is essential for large-scale SCP production. By establishing optimal conditions, the yield and nutritional quality of SCP can be significantly enhanced [132]. For example, a study by [133] investigated a novel method for producing SCPs using the fungal strain *Paradendryphiella arenariae* PG1 and Bengal gram husk, an agricultural waste product. Through careful optimization and monitoring of key factors such as pH, temperature, and fungal growth over a 72-h period, they achieved a crude protein content of 54.56%. This study underscores the importance of controlled environmental factors, such as fermentation conditions, in enhancing the nutrient content of SCPs, leading to improved protein yield in bioprocesses.

### 5.2. Genetic Engineering

The genetic and metabolic engineering of microbial strains is vital for enhancing the nutrient content of SCPs. This methodology involves modifying microbial genomes to increase the production of essential nutrients such as amino acids, vitamins, lipids, and minerals [134,135,136]. For instance, *Schizochytrium* and *Chlamydomonas reinhardtii* have been genetically engineered to enhance their nutritional quality by overexpressing targeted genes and inhibiting competing pathways [137,138]. Similarly, metabolic engineering strategies, such as the modification of metabolic pathways in *Methylococcus capsulatus* MIR to produce glycogen-deficient mutants, have been employed to optimize SCP production for increased nutritional yield [139].

### 5.3. Substrate Optimization

Substrate optimization is pivotal in SCP biofortification. By incorporating specific nutrients into fermentation media, such as selenium, the microbial biomass can be enriched, thereby improving the nutritional content of the SCPs. The use of selenium-enriched yeast, as demonstrated in the study by [130], enhances the nutritional quality of SCPs by providing an essential trace element that supports fish growth and enhances stress resilience. Furthermore, a study by [140] showcased the production of SCP rich in potassium using *Nectaromyces rattus* cultivated on biogas slurry and molasses, illustrating how substrate selection can influence the nutritional profile of SCPs.

### 5.4. Selective Strain Development

Another critical biofortification strategy involves the development of selective strains with naturally superior nutrient content. According to [141], *M. capsulatus* contains approximately 70% protein on a dry mass basis. Strains of *M. capsulatus* MIR have been identified and modified to produce higher protein content, as demonstrated by [139]. Such strains contribute to improved SCP production, rendering them a more efficient and sustainable protein source for aquafeeds.

### 5.5. Metabolic Pathway Optimization

Metabolic pathway optimization entails modifying the biochemical pathways of microorganisms to enhance the synthesis of key nutrients. This approach has been successfully applied to microalgae to promote the production of essential fatty acids, amino acids, and other vital nutrients. Genetic and metabolic modifications in microalgal species such as *Schizochytrium* sp. and *C. reinhardtii* have been utilized to improve their nutritional profiles, particularly for aquaculture applications [142].

### 5.6. Epigenetic Modulation

Epigenetic modulation offers a less invasive approach to influencing nutrient synthesis in SCPs. By adjusting environmental conditions such as light, temperature, and nutrient availability, the synthesis of key nutrients can be modulated without altering the DNA sequence [143]. This approach provides an alternative to genetic manipulation, presenting a more sustainable method to enhance the nutritional quality of SCPs.

### 5.7. Co-Cultivation Systems

Co-cultivation systems, wherein different microbial species are grown together, exploit symbiotic interactions to enhance the yield and nutritional quality of SCPs [30,144]. For example, co-culturing bacteria with microalgae can result in SCPs with balanced amino acid and fatty acid profiles, rendering them more nutritionally complete and better suited for inclusion in aquaculture diets. Historically, SCP production relied on monoculture systems, particularly during and after World War I, primarily for animal feed [145]. However, advancements in biotechnology have shifted the focus toward co-culture systems due to their superior efficiency and enhanced nutritional benefits compared to monocultures [145]. Mixed-culture fermentation has emerged as a key approach in co-cultivation, yielding significant improvements in SCP production. For instance, the co-culture of *Ganoderma lucidum* and *Candida utilis* produced 16.23% more protein than their respective monocultures [146]. Similarly, co-cultivating *C. utilis* and *A. niger* increased the protein content of dried pomace to 20%, demonstrating the potential of co-culture systems to enhance nutrient profiles [147].

### 5.8. Cell Wall Disruption

A significant challenge associated with microalgae-based SCPs is the presence of a robust cell wall, which limits the bioavailability of nutrients [148]. To address this issue, techniques such as mechanical disruption, enzymatic hydrolysis, and chemical treatments are employed to break down the cell wall, thereby enhancing nutrient accessibility and improving overall yield [149].

## 6. Production of SCP

**Figure 4:**
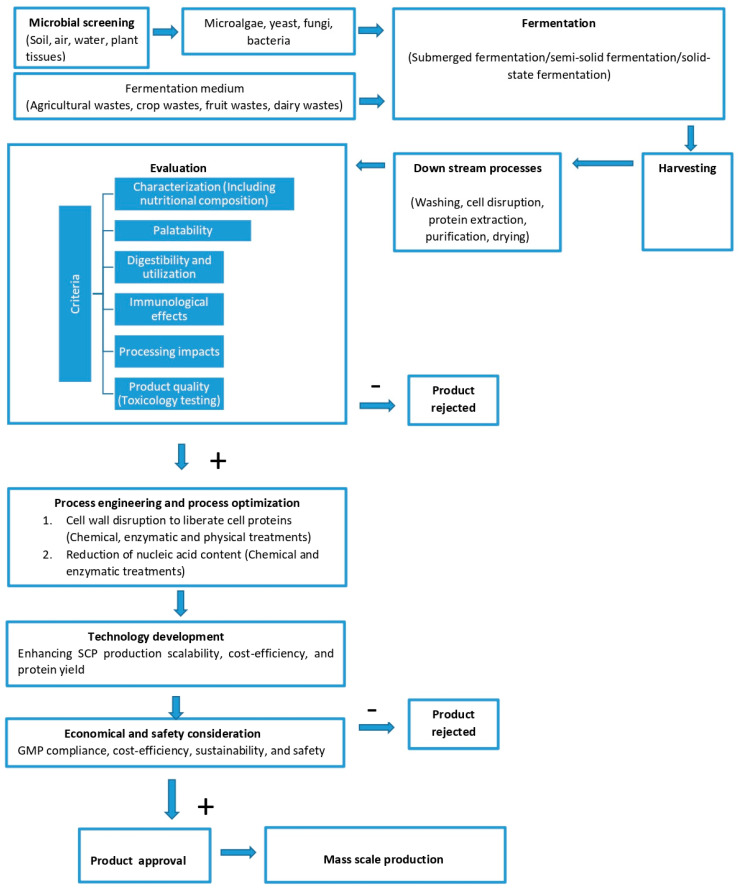
Flow chart of SCP production and evaluation. Modified based on [24,155,158]. GMP: Good Manufacturing Practice.

The production of SCPs encompasses several critical steps, including microbial screening, fermentation, harvesting, and downstream processing (Figure 4). The process initiates with microbial screening, wherein strains are isolated from various sources, including soil, air, water, and plant tissues (stem, leaf, fruit, flower, root) [26]. Techniques from [150,151] are employed for the isolation of endophytic SCPs. The selection of suitable microorganisms is typically facilitated through mutagenesis and advanced genetic techniques [152], while genetic profiling and omics technologies are utilized to identify strains exhibiting beneficial characteristics [153,154].

The subsequent step, fermentation, is a critical phase that is primarily categorized into submerged, semi-solid, and solid-state fermentation [26]. Submerged fermentation employs liquid nutrient-rich media (e.g., molasses, fruit juices) and necessitates aeration and cooling to manage heat generated during the fermentation process. Continuous operation and harvesting are facilitated through centrifugation or filtration. Semi-solid fermentation represents a modification of solid-state fermentation, integrating additional free liquid to enhance nutrient availability and process control. Conversely, solid-state fermentation utilizes solid substrates, such as wheat bran or rice, and is conducted in temperature-controlled environments over several days. The optimization of substrates, alongside careful supplementation of carbon, nitrogen, and phosphorus, is paramount for enhancing biomass yield and reducing fermentation time [155].

Following fermentation, the biomass undergoes harvesting, wherein microbial biomass is separated using organism-specific techniques. This is succeeded by downstream processing, which includes washing, cell disruption, protein extraction, and purification [156]. These processes are vital for isolating purified proteins from the microbial biomass, despite variations in initial composition and extractability [157]. Upon extraction, the proteins are dried, packaged, and prepared for shipment. The entire process encompasses comprehensive steps of propagation, fermentation, harvesting, and final product refinement, ensuring the production of SCPs suitable for applications such as aquaculture feeds.

## 7. Criteria for the Selection and Evaluation of SCPs in Aquaculture

The selection of microbial strains for SCP production is influenced by multiple factors, including oxygen and heat requirements, growth rate, yield, pH and heat tolerance, genetic stability during fermentation, protein composition, and the ease of recovery and purification [155]. Genetic profiling and omics technologies assist in identifying strains with advantageous characteristics for high-quality protein production [153,154]. This selection process is followed by the optimization of fermentation conditions, encompassing substrate choice, carbon and nitrogen supplementation, and temperature control, to maximize biomass yield and minimize fermentation time [155].

The subsequent stage involves evaluating SCPs based on a standardized set of criteria, focusing on nutritional response parameters as outlined by [24]. This evaluation is conducted through a seven-step process: characterization (including nutritional composition such as protein concentration, amino acid profile, lipid content, carbohydrates, vitamins, and minerals), palatability, digestibility, utilization, immunological effects, processing impacts, and product quality (Figure 4). These criteria facilitate informed decision making regarding the suitability of SCP ingredients for aquaculture [24].

Process engineering constitutes a crucial aspect during the optimization of SCP production, encompassing steps such as cell wall disruption and the reduction of nucleic acids, which enhance the efficiency of protein extraction and improve overall yield. Moreover, technology development plays a vital role in enhancing the scalability of SCP production, reducing costs, and increasing protein recovery [155].

When considering SCP as a protein source for aquafeeds, a meticulous evaluation of production costs is essential, as feed costs represent a significant portion of aquaculture expenses [8]. The development of cost-effective SCP-based feeds could enhance the economic feasibility of the industry [63]. Furthermore, ensuring the safety of SCPs for both human and animal consumption is imperative. This necessitates rigorous monitoring to prevent harmful toxins produced by specific microorganisms, as well as adherence to regulatory standards, product approvals, and licensing [155].

In the context of large-scale SCP production, the implementation of good manufacturing practices (GMPs) is essential. GMP ensures consistent product quality, maintains regulatory compliance, and addresses safety concerns. Key aspects of GMP include raw material quality, controlled fermentation conditions, hygiene, and proper post-production handling. These practices are vital for ensuring safe, reliable, and consistent SCPs suitable for use in commercial aquaculture feeds.

## 8. Effects of SCPs in Aquaculture

**Figure 5:**
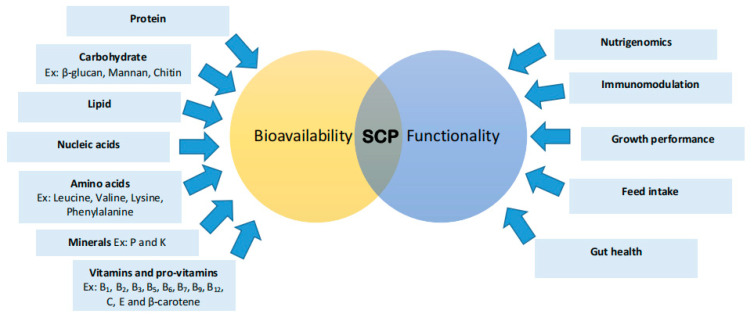
Nutritional Value and Functionality of Single-Cell Proteins (SCPs) in Aquafeeds. This diagram illustrates the bioavailability and functional roles of SCPs, highlighting key nutritional components such as protein, carbohydrates (e.g., β-glucan, mannan, chitin), lipids, nucleic acids, amino acids (e.g., leucine, valine, lysine, phenylalanine), minerals (e.g., phosphorus (P) and potassium (K)), and vitamins and provitamins (e.g., B_1_ (thiamine), B_2_ (riboflavin), B_3_ (niacin), B_5_ (pantothenic acid), B_6_ (pyridoxine), B_7_ (biotin), B_9_ (folate), B_12_ (cobalamin), C (ascorbic acid), E (tocopherol), and β-carotene). Functionally, SCPs contribute to nutrigenomics, immunomodulation, growth performance, feed intake, nutrient digestibility, and gut health.

This section examines the multifaceted roles of SCPs in aquaculture, emphasizing their specific impacts on gene expression, immunomodulation, feed intake and growth performance, and gut health in farmed species (Table 3) (Figure 5). A comprehensive understanding of these effects elucidates the potential of SCPs to enhance aquaculture efficiency and sustainability.

### 8.1. Nutrigenomic Effects of SCP

**Figure 6:**
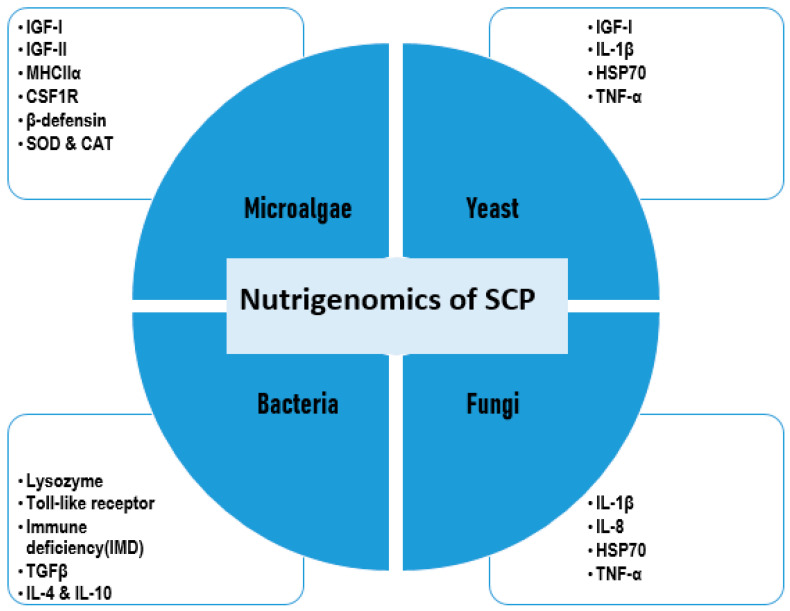
Nutrigenomic effects of different types of single-cell proteins (SCPs). Sources: [58,97,165,173,178,181,182]. This diagram illustrates some nutrigenomic impacts of SCPs on various immune and growth-related molecular markers in fish (IGF-I: Insulin-like Growth Factor I, IGF-II: Insulin-like Growth Factor II, MHCIIα: Major Histocompatibility Complex Class II alpha chain, CSF1R: Colony Stimulating Factor 1 Receptor, β-defensin: Beta-Defensin, SOD: Superoxide Dismutase, CAT: Catalase, IL-1β: Interleukin-1 Beta, HSP70: Heat Shock Protein 70, TNF-α: Tumor Necrosis Factor Alpha, TGFβ: Transforming Growth Factor Beta, IL-4: Interleukin-4, IL-8: Interleukin-8, IL-10: Interleukin-10).

Recent advancements are uncovering the molecular mechanisms underlying dietary effects and individual variations, providing critical insights for optimizing nutrition in aquaculture species (Figure 6). Nutritional genomics (nutrigenomics), which investigates how diet influences gene expression, plays a pivotal role in promoting health and preventing diseases [159]. By integrating genomic research, biotechnology, and molecular nutrition, nutrigenomics drives innovation in the field, refining nutritional strategies [160]. Furthermore, it facilitates the development of personalized diets tailored to an individual’s genetic profile, thereby enhancing health outcomes [160,161].

Nonetheless, a significant challenge in nutritional research arises from the complex and diverse nature of foods and their nutritional components. Each nutrient interacts with multiple target sites, exhibiting varying levels of affinity and specificity [162]. Nutrition substantially influences gene activity, regulating gene activation and suppression [163]. Nutrients primarily affect gene expression through transcription factors that interact with DNA and govern specific response elements [164]. Additionally, co-repressor and co-activator proteins play a crucial role in gene regulation, underscoring their importance in modulating co-activator expression [164].

Research on potential SCP sources, such as *S. cerevisiae*, has demonstrated their capacity to influence gene expression in fish. Specifically, supplementation with 4 g/kg of *S. cerevisiae* in sea bream diets significantly regulated the expression of genes involved in stress response (heat shock proteins (HSP70)), growth (IGF1), and immune function (IL-1β), underscoring the potential of SCP sources to modulate genetic pathways related to health and disease resistance in aquaculture species [165]. Heat shock proteins (HSPs) are essential intracellular proteins that aid organisms in coping with various stresses, including environmental factors such as elevated temperatures, oxygen deficiency, and heavy metals, as well as biological threats from pathogens [166]. Insulin-like growth factor-I (IGF-I) is implicated in numerous roles in fish, including promoting DNA synthesis, protein production, and cartilage development, in addition to enhancing adaptability to seawater [167]. Furthermore, IGF-I is involved in reproductive processes, such as stimulating spermatogenesis and final oocyte maturation [167]. Interleukin-1β (IL-1β) is a pivotal pro-inflammatory cytokine produced by various cells of the innate immune system, including blood monocytes, macrophages, and neutrophils [168,169]. IL-1β plays a critical role in antiviral and antibacterial defense, including its involvement in the immune response against nervous necrosis viruses in the orange-spotted grouper (*Epinephelus coioides*) [170,171,172].

Feeding *Litopenaeus vannamei* a diet partially substituted with *Arthrospira platensis* (Spirulina) significantly enhances the expression of IGF-I and IGF-II genes [173]. These insulin-like growth factors are critical polypeptides involved in promoting growth, immune modulation, and overall physiological regulation in fish [174,175,176]. Pacific white shrimp (*L. vannamei*) fed diets containing SCP derived from *Methylophilus capsulatus* as a fish meal replacement exhibited increased expression of immune-related genes, including lysozyme, Toll-like receptor, and immune deficiency (IMD) [97].

Incorporating ImmunoWall^®^ (a yeast-based prebiotic produced by ICC Brazil) into fish diets significantly enhances immune responses by upregulating proinflammatory cytokines and immune-related genes, including TNF-α and IL-1β [58]. Tumor necrosis factor (TNF-α) is a pivotal proinflammatory cytokine essential for both innate and adaptive immunity, as it activates various immune cells and stimulates the release of additional cytokines to facilitate infection clearance [177]. Fish fed with *Nannochloropsis gaditana* and *Tetraselmis chuii* (microalgae) demonstrated upregulation of immune-related genes such as Major Histocompatibility Complex Class II alpha chain (MHCIIα), Colony Stimulating Factor 1 Receptor (CSF1R), and β-defensin [178]. Dietary supplementation with *S. platensis* significantly upregulated the expression of antioxidant genes superoxide dismutase (SOD) and catalase (CAT) in the liver of rainbow trout, indicating its potential role in enhancing oxidative stress defense [179].

FeedKind^®^, developed by Calysta, is composed of *M. capsulatus* bacterial meal. Supplementation of feed with FeedKind^®^ has been shown to influence gene expression in spotted sea bass (*L. maculatus*) [180]. In this context, anti-inflammatory genes such as Transforming Growth Factor Beta (tgfβ), interleukin-4 (IL-4), and interleukin-10 (IL-10) were expressed in the intestine, underscoring its potential to modulate immune-related pathways. Dietary supplementation with *A. oryzae* resulted in enhanced gene expression in Nile tilapia, notably increasing mRNA levels of IL-1β and interleukin-8 (IL-8) in the liver under varying salinity conditions [181]. Diets supplemented with *A. oryzae* also led to the upregulation of immune-related genes, such as TNF-α, IL-1β, and HSP70, in Nile tilapia [182]. Overall, the application of SCPs in aquaculture diets underscores their potential to enhance health and stress resilience through targeted gene regulation.

### 8.2. Effects of SCPs on Immunomodulation

The yeast cell wall comprises several components, including β-glucan, mannan, protein, lipid, and chitin, with β-glucan constituting the largest proportion, ranging from 29% to 64% [183,184,185]. β-glucans, widely utilized as prebiotics, are natural compounds found in the cell walls of yeast, algae, plants, fungi, and bacteria. These compounds consist of a D-glucose backbone with side chains linked by β-glycosidic bonds, and their structural variations can induce differing immune responses [186,187]. Specifically, β-glucan derived from yeast and fungi has been demonstrated to regulate and enhance immune responses [186]. For instance, a study by [188] found that yeast β-glucan enhances respiratory burst activity in macrophages of Atlantic salmon (*S. salar*). The Dectin-1 receptor, prevalent on immune cells such as dendritic cells, neutrophils, eosinophils, macrophages, monocytes, and certain T-cells, plays a critical role in the immune response [186]. Research by [189] also suggests that Atlantic salmon macrophages may possess a specialized receptor for yeast glucan. When β-glucan interacts with the Dectin-1 receptor, it triggers intracellular signaling pathways that activate the NF-κB transcription factor, promoting cytokine release, phagocytosis, and the generation of reactive oxygen species [186].

Mannan oligosaccharides (MOSs), derived from yeast cell walls, support gut health and digestion by preventing pathogens from binding to glycoprotein receptors [190]. MOS can enhance the immune status of various fish species, including rainbow trout and European sea bass (*Dicentrarchus labrax*) [191,192]. Furthermore, MOS from yeast cell walls enhances immune modulation in aquaculture by blocking pathogen colonization, strengthening the epithelial barrier, boosting immunity, improving growth and feed efficiency, and increasing disease resistance [193]. Yeast β-1,3 and β-1,6 glucans also enhance immune modulation in rainbow trout (*O. mykiss*) by boosting macrophage bactericidal activity and reactive oxygen species production, aiding in combating both avirulent and virulent strains of *Aeromonas salmonicida* [194].

The Protec™ feed, developed by Skretting and enriched with β-glucans, has been shown to enhance immunity in rainbow trout by increasing leukocyte respiratory burst activity and the production of specific immunoglobulin M (IgM) against *Lactococcus garvieae* [195]. In a study involving freshwater prawn (*Macrobrachium rosenbergii*), diets containing 2–8% *Chlorella vulgaris* improved immune responses, as evidenced by increased prophenol oxidase activity and total hemocyte counts following a bacterial challenge with *Aeromonas hydrophila*, highlighting the immunomodulatory potential of microalgae as a single-cell protein [196]. A recent investigation on golden pompano (*Trachinotus ovatus*) demonstrated that incorporating 3% *Schizochytrium* sp. into the diet enhanced non-specific immune responses [197].

Chitin, mannoprotein, and glucan from the cell wall of *S. cerevisiae* are recognized for stimulating the immune system and supporting growth in sea bream [198]. Including *S. cerevisiae* in diets can enhance fish immunity by significantly increasing lysozyme activity, phagocyte activity, and immunoglobulin M (IgM) levels [165]. ImmunoWall^®^, a commercial prebiotic rich in yeast β-glucan and MOS, has been shown to reduce mortality rates in fish following infections with *Lactococcus garvieae* and *Aeromonas hydrophila* when administered orally [58]. MOS enhances immune responses by stimulating the liver to produce mannose-binding proteins that target bacteria, support the growth of beneficial gut bacteria, suppress harmful pathogens, and prevent pathogen attachment by blocking glycoprotein receptors [58,199]. Moreover, β-glucan enhances zebrafish resistance to spring viremia of carp virus infection (SVCV) by activating the type I interferon (IFN) antiviral immune response following viral infection [200]. Additionally, β-glucan derived from baker’s yeast is known to bolster immune responses [201]. Unibio, a sustainable protein company, developed Uniprotein^®^, which demonstrated improved disease resistance and potential immunomodulatory effects in rainbow trout when utilized as a partial fishmeal replacement [96]. Microalgal products, particularly those derived from *Schizochytrium* SCP, have been shown to enhance immune function in fish, as evidenced by increased goblet cell proliferation, mucus production, and inducible nitric oxide synthase activity in Atlantic salmon (*S. salar*) [202]. Finally, studies on Pacific white shrimp (*P. vannamei*) fed diets containing SCP derived from *M. capsulatus* resulted in a more robust innate immune response compared to those fed the control diet [97].

### 8.3. Effect of SCPs on Feed Intake and Growth Performance

The effects of SCPs on growth and feed intake have been extensively studied across various aquaculture species. In a study by [203], the impact of *Nannochloropsis* SCP on the growth performance of post-larval *Marsupenaeus japonicus* was evaluated, revealing that higher inclusion levels of SCP (up to 70 g/kg) improved growth and feed utilization. This finding aligns with research suggesting that the palatability of microalgal SCP significantly influences growth performance, as the acceptance of the feed by the target species directly affects feed intake and overall growth efficiency [24]. For instance, ref. [204] observed improved feed intake when replacing 25% of corn gluten meal with Algamaxx (derived from *Spirulina*), emphasizing the role of SCP in enhancing feed consumption.

Similarly, a study by [205] demonstrated that the incorporation of a 10% Spirulina-based algal meal into the diet of rainbow trout resulted in increased final weight, average daily gain, and specific growth rate. Furthermore, research on Pacific white shrimp revealed that a diet comprising approximately 60% *Candida utilis* resulted in enhanced growth rates without any adverse effects when compared to a diet exclusively based on fishmeal [206]. Previous investigations have indicated that *Candida utilis* is a more suitable source of single-cell protein (SCP) than *S. cerevisiae* for both salmon and shrimp diets [20].

Additionally, research conducted by Akvaforsk and the Aquaculture Protein Centre in Norway indicated that substituting fishmeal with FeedKind Aqua protein improved the growth performance of Atlantic salmon, underscoring the potential of SCPs in aquaculture. Similarly, Nile tilapia (*Oreochromis niloticus*) exhibited significant growth when fed a diet containing 10% Novacq™ (marine microbial biomass) and 0% fishmeal [207]. Enhanced growth was also observed in post-larvae and juvenile *P. vannamei* when provided with a diet containing Novacq™ [51]. Collectively, these findings suggest that SCPs can effectively enhance growth performance and feed intake across various aquaculture species.

### 8.4. Effect of SCPs on Gut Health

The type of diet has a significant impact on the structure and diversity of bacterial communities within the gastrointestinal tract, with these microbial populations playing a critical role in maintaining host health by modulating immune function [208]. SCPs, including those derived from microalgae and yeast, have demonstrated positive effects on gut health by promoting the growth of beneficial bacteria and enhancing microbiome diversity. For example, the inclusion of *Schizochytrium* SCP in the diet of rainbow trout (*O. mykiss*) increased microbiome diversity within the gastrointestinal tract, highlighting its potential role in improving gut health and microbial composition [208]. Similarly, yeast-derived products have exhibited beneficial effects on intestinal health. Yeast cells provide energy substrates that support intestinal cell function, while dietary nucleotides from brewer’s yeast have been shown to facilitate rapid intestinal repair, enhance gut flora, and improve mucosal surfaces, potentially leading to the elongation of the intestinal tract in aquatic animals [209,210]. Furthermore, mannose-binding proteins derived from yeast SCPs can target bacteria, support the proliferation of beneficial gut bacteria, inhibit harmful pathogens, and prevent pathogen attachment by obstructing glycoprotein receptors [58,199].

Moreover, bacterial meal (BM) derived from natural gas has been demonstrated to mitigate soybean-meal-induced enteritis in Atlantic salmon by promoting intestinal health and diminishing inflammation in the distal intestine [211]. Calysta’s FeedKind^®^ protein, another SCP, is asserted to contribute to a healthy gut in both Atlantic salmon and rainbow trout [180]. These findings underscore the increasing recognition of SCPs as beneficial components in aquaculture diets, with the capacity to enhance gut health and overall immune function.

**Table 3:** Effects of SCPs in aquaculture.

Type of SCP	Test Organism	Size/Stage	Duration	Concentration	Result	Reference
*A. niger*	*Fingerlings* *Cirrhinus reba*	Fingerlings 4.66 ± 0.2 g	60 days	10% and 20%	- Improved growth performance - Carcass analysis revealed that SCP-fed fingerlings exhibited a balanced amino acid and fatty acid profile	[61]
*Filamentous fungi* *Paecilomyces variotii*	*Atlantic Salmon* *(S. salar)*	24 g	63 days	20%	- Upregulation of biomarkers: ifng, IL-10, tgfb, arg1, inducible nitric oxide synthase (inos), peroxiredoxin (prx; 1.41), superoxide dismutase (SOD; 0.25), forkhead box P3 (foxp3; 0.43), and interferon regulatory factor 5 (irf5; 0.38).	[212]
*Bacterial protein meal (BPM)*	*Atlantic Salmon* *(S. salar)*	170 g	48 days	18% and 36%	- Increased specific growth rate	[213]
*S. cerevisiae (whole yeast cell)*	*Oreochromis niloticus*	80 ± 5 g	2 months	Basal diet + 2 g/kg	- Improved growth performance and non-specific immunity	[57]
*Immunowall * *S. cerevisiae (yeast) β-glucan (βG) and mannan oligosaccharides (MOSs)*	*Oreochromis niloticus*	50.7 ± 0.8 g	2 months	0.2%	- Improved growth performance, non-specific immune responses (phagocytic activity, phagocytic index and lysozyme activity), and gene expressions (TNF-α and IL-1β)	[58]
*Microalgae* *S. pacifica*	*Rainbow trout * *(O. mykiss)*	14.66 g	35 days	10% Spirulina meal + basal diets	- Increased final weight, average daily gain, and specific growth rate - Carcass weight and consumable yield of the rainbow trout were higher	[205]
*Microalgae meal*	*Juvenile Pacific white shrimp* *(L. vannamei)*	Juveniles 1.73 ± 0.003 g	44 days	10%, 20%, 30%, and 40%	- Produced shrimp with more intense red/orange color and significantly higher total carotenoid concentration than the control diet	[214]
*Tisochrysis lutea and Tetraselmis suecica*	*Gilthead seabream* *(S. aurata)*	49 ± 0.4 g	84 days	Replaced 10% crude protein from the mixture of vegetable protein sources in the control diet	- Improved skin pigmentation	[15]
*S. cerevisiae SCP*	Atlantic salmon *(S. salar)*	28 g	89 days	Substituting 40% of the crude protein from fishmeal	- Increased average daily feed intake and feed conversion ratio - Lower digestibility of crude protein	[46]
*Brewer’s yeast * *S. cerevisiae*	*Juvenile sea bass * *(Dicentrarchus labrax)*	Juveniles 12 g	84 days	10%, 20%, 30% of dietary nitrogen from yeast	- Improved feed efficiency	[215]
*S. maxima*	*Oreochromis mossambicus* fry	279 mg (20–30 days old, mixed sexes)	63 days	Fishmeal was replaced with algae protein at ratios of 20% and 40%	- Improved growth rate and protein utilization	[216]
*Brewer’s yeast * *(S. cerevisiae)*	Juvenile Thai panga *(Pangasianodon hypophthalmus × Pangasius bocourti)*	Juveniles 36.4 ± 0.07 g	270 days	45%	- Improved lysozyme activity and total immunoglobulin (Ig) - Improved growth performance and immune response	[217]
*SCP methanophillic bacterial origin*	Rainbow trout *(Salmo guirdnerii Richardson)*	16–17 g	112 days	21% dry weight	- Improved growth	[218]
*Methylococcus capsulatus* *bacteria meal*	Pacific white shrimp *(L. vannamei)*	0.88 ± 0.01 g	49 days	45% replacing fish meal	- Intestinal circular muscle layer thickness was significantly increased - Increased height of mucosal folds	[219]
*Corynebacterium glutamicum SCP*	Flathead grey mullet *(Mugil cephalus)*	68 g	113 days	10%, 20%	- Modulation of gut microbiota	[220]

## 9. Challenges and Future Perspectives

The utilization of single-cell proteins (SCPs) in aquaculture feeds presents considerable potential; however, it is accompanied by significant challenges that must be addressed to facilitate industry advancement [20,155]. A primary obstacle to the adoption of SCPs in the aquafeed sector is the elevated production cost, which currently restricts their widespread use [24]. The production of SCP necessitates advanced bioreactors, sterile environments, and energy-intensive downstream processing [152]. The employment of low-cost substrates, such as agro-industrial waste, along with energy-efficient technologies, could mitigate costs, while process integration and continuous fermentation may improve economic viability [18,155].

Moreover, SCP production is limited by technical challenges associated with fermentation technologies, contamination management, and downstream processing [155]. Achieving optimal growth conditions for various microorganisms necessitates precise control of parameters such as pH, temperature, and oxygen levels [133]. Advances in bioreactor design, automation, and real-time monitoring are critical for scaling production.

Another significant limitation for the large-scale application of SCP is low production yields. Despite rapid growth rates, biomass yield per unit substrate often proves inadequate for the commercial replacement of fishmeal. Genetic engineering and metabolic optimization may enhance yields, while co-cultivation strategies could further augment productivity [137,138,139,145].

The nutritional profiles of SCPs can exhibit significant variability due to seasonal fluctuations, species differences, and culture conditions. Implementing standardized cultivation protocols and real-time nutrient monitoring can contribute to uniformity.

Digestibility challenges persist as a substantial barrier to SCP utilization. For example, the marine microalga *N. gaditana* demonstrates limited nutrient accessibility, adversely affecting its digestibility in juvenile Nile tilapia [221]. Mechanical and physical treatments, such as microfluidization, have been shown to enhance nutrient accessibility, thereby improving the overall digestibility and utility of SCP in aquafeeds. Continued research and innovation in these technologies are imperative.

Another critical concern is the potential production of harmful metabolites by certain microorganisms employed in SCP production. Strains such as *A. parasiticus* and *A. flavus* may generate mycotoxins, posing health risks to both animals and humans [71]. Similarly, cyanobacteria can produce cyanotoxins with detrimental effects [222]. Addressing this issue necessitates meticulous strain selection, bolstered by advanced genetic and biochemical screening techniques. Furthermore, rigorous toxicological assessments, encompassing both acute and chronic toxicity studies across various species, should be instituted as a standard practice in SCP production to ensure safety [34].

The elevated nucleic acid content (6–10%) of SCPs presents another challenge, as it may lead to increased serum uric acid levels, resulting in health complications such as gout and kidney stones [26]. While chemical and enzymatic methods for nucleic acid reduction are available, they often incur substantial costs and may compromise the nutritional quality of the final product. Developing cost-effective and environmentally sustainable alternatives, such as selective breeding of low-nucleic-acid strains or optimizing fermentation conditions, could provide viable solutions.

Palatability and processing inconsistencies further hinder the utilization of SCPs in aquafeeds. Research on *M. extorquens* SCP inclusion in rainbow trout diets indicated reduced feed intake and weight gain attributable to suboptimal palatability [55]. This issue may be addressed through the integration of natural palatability enhancers or feed attractants, facilitating higher inclusion rates without negatively affecting growth performance. Additionally, the processing conditions for SCP significantly impact product quality, necessitating advancements in production technologies to ensure consistency and reliability [88].

The structural characteristics of certain SCPs, including thick cell walls, obstruct nutrient absorption and digestibility [47]. Techniques such as cell wall disruption via microfluidization have been effective, as demonstrated with *C. vulgaris*, where the bioavailability of essential nutrients improved without compromising overall quality [223]. Scaling up these techniques for industrial application could substantially enhance the utility of SCPs in aquafeeds.

Biofortification strategies, encompassing production process optimization, genetic engineering, substrate optimization, selective strain development, metabolic pathway optimization, epigenetic modulation, co-cultivation, and cell wall disruption, hold significant promise for enhancing the nutritional value of SCPs. These methodologies can be applied to improve the quality and sustainability of aquafeeds. As research progresses, these strategies present a hopeful outlook for the biofortification of SCPs within aquaculture.

## 10. Conclusions

SCPs represent a transformative and sustainable alternative to traditional protein sources in aquafeeds, addressing the urgent necessity to diminish reliance on fishmeal. With their potential for biofortification and nutrigenomic advantages, SCPs can significantly enhance growth performance, immune modulation, and gut health in aquaculture species. Furthermore, the capability of microorganisms to convert low-cost agricultural and industrial by-products into high-quality protein aligns with circular economy principles and promotes environmental sustainability.

Despite these promising benefits, challenges such as enhancing nutrient bioavailability, reducing nucleic acid content, and achieving cost-effective large-scale production must be addressed to fully realize the potential of single-cell proteins (SCPs) in aquaculture. Overcoming these obstacles necessitates a multidisciplinary approach that integrates biotechnological innovations, cost-efficient processing methods, and comprehensive safety evaluations. By addressing these barriers, the SCP industry can maximize its potential, thereby promoting sustainable growth in aquaculture. Future research should prioritize the optimization of SCP production processes, the exploration of novel sources such as endophytes, and the elucidation of the molecular mechanisms underlying their immunomodulatory and nutrigenomic effects. Addressing these gaps will facilitate the full integration of SCPs as functional feed ingredients, thus transforming aquaculture practices and contributing to global food security and environmental resilience.

## Figures and Tables

**Table 1:** Different types of SCPs used in aquaculture, indicating their protein content, characteristics, and challenges.

Type of SCP	Examples	Crude Protein Content (%)	Fish Used	Characteristics	Challenges	Source(s)
Microalgae	*Spirulina* spp., *Chlorella vulgaris*, *Desmodesmus* sp.	60–70	*Oreochromis niloticus*, *Clarus gariepinus*, *Salmo salar*	- Production of omega-3 fatty acid	- Low digestibility	[18,25]
Yeast	*Saccharomyces cerevisiae*, *Candida utilis*, *Kluyveromyces marxianus*	45–65	*Oncorhynchus mykiss*, *Penaeus vannamei*, *Salvelinus alpinus*, *Perca fluviatilis*, *S. salar*	- Easily harvested - High lysine and malic acid content - Low nucleic acid content - Production of - vitamins and - micronutrients	- Low growth rate - Low methionine and protein content	[26]
Fungi	*Aspergillus oryzae*, *Yarrowia lipolytica*, *Myrothecium verrucaria*	30–45	*O. niloticus*, *L. vannamei*, *S. salar*	- Rich in lysine and threonine - Low nucleic acid content	- Enhancing protein content and essential amino acid profile	[18,27]
Bacteria	*Methylococcus capsulatus*, *Methylobacterium extorquens*, *Cupravidus necator*	50–80	*L. vannamei*, *Lateolabrax maculatus*, *S. salar*, *O. mykiss*, *Seriola quinqueradiata*	- Short generation time - Rapid growth - High protein content	- High nucleic acid content - Production of toxins - Low palatability	[18,28]

**Table 2:** Major SCP-producing countries and key companies.

Company	Country	Product	Type of SCP	Target Species	Administration Method	Focus Areas
Calysta	USA	FeedKind Aqua^®^	*M. capsulatus*	Shrimp, salmon, and warm water carnivorous finfish	Feed ingredient	- Enhance gut health and immunity
Calysta	USA	FeedKind Terra^®^	*M. capsulatus*	Piglet and chick	Starter diets (early animal nutrition)	- Improve growth rate and feed intake
KnipBio	USA	KnipBio Meal (KBM)	*M. extorquens*	Salmon, shrimp	Feed ingredient	- Maintain health and promote growth
Arbiom	USA	Yusto	Yeast protein	Human	Sublimate all types of foods such as animal-free meat and dairy products, snacks, sauces, and specialized nutrition products, food ingredient	- Support good digestion and intestinal microbiota
Leiber GmbH	Germany	CeFi^®^ pro	Brewer’s yeast	Fish and shrimp	Feed ingredient	- Stimulate the metabolism - Enhance feed intake, performance, and gut health
Algenuity	UK	Chlorella	*Chlorella* sp.	Human	As a food ingredient	- Naturally contain antioxidants, beta-glucan, vitamins, minerals, and healthy plant-based omega-6 oils
Unibio	Denmark	Uniprotein^®^	*Methanotrophic* bacteria	Pig, salmon, trout, shrimp, sea bream, sturgeon	Feed ingredient	- Promoting growth and bolstering the immune system
Lallemand	Canada	Lyfe^®^, Engevita^®^, Lake States^®^, Toravita^®^, Bakon^®^, and Lalvita^®^	Baker’s and brewer’s yeast	Human	Food ingredient	- Enrich the nutritional profile - Contribute to better emulsification, taste intensity, and longer-lasting flavor perception

## Data Availability

The original contributions presented in this study are included in the article. Further inquiries can be directed to the corresponding author.
